# The *N*^6^-methyladenosine demethylase ALKBH5 negatively regulates the osteogenic differentiation of mesenchymal stem cells through PRMT6

**DOI:** 10.1038/s41419-021-03869-4

**Published:** 2021-06-04

**Authors:** Zhaofeng Li, Peng Wang, Jinteng Li, Zhongyu Xie, Shuizhong Cen, Ming Li, Wenjie Liu, Guiwen Ye, Guan Zheng, Mengjun Ma, Shan Wang, Wenhui Yu, Yanfeng Wu, Huiyong Shen

**Affiliations:** 1 Department of Orthopedics, The Eighth Affiliated Hospital, Sun Yat-sen University, 3025# Shennan Road, Shenzhen, 518033 People’s Republic of China; 2 Department of Orthopedics, Sun Yat-sen Memorial Hospital, Sun Yat-sen University, 107# Yan Jiang Road West, Guangzhou, 510120 People’s Republic of China; 3 Center for Biotherapy, The Eighth Affiliated Hospital, Sun Yat-sen University, 3025# Shennan Road, Shenzhen, 518033 People’s Republic of China

**Keywords:** RNA modification, Mesenchymal stem cells, Stem-cell differentiation

## Abstract

*N*^6^-methyladenosine (m^6^A) modification is widespread in messenger RNAs and increasing evidence suggests the crucial roles of m^6^A in cell differentiation and tissue development. However, whether m^6^A modulates the osteogenic differentiation of mesenchymal stem cells (MSCs) has not been fully elucidated. Here we show that conditional knockout of the demethylase *Alkbh5* in bone marrow MSCs strengthened bone mass in mice. Loss- and gain-of-function studies demonstrated that ALKBH5 negatively regulates the osteogenic differentiation of MSCs in vitro. At a mechanistic level, meRIP-seq and RNA-seq in MSCs following knockdown of ALKBH5 revealed changes in transcripts of *PRMT6* containing consensus m^6^A motifs required for demethylation by ALKBH5. Furthermore, we found that ALKBH5 accelerates the degradation rate of *PRMT6* mRNA in an m^6^A-dependent manner, and that the ALKBH5-PRMT6 axis regulates the osteogenesis of MSCs, mainly through activation of the PI3K/AKT pathway. Thus, our work reveals a different facet of the novel ALKBH5-PRMT6 axis that modulates the osteogenic differentiation of MSCs, which can serve as a target to improve the clinical use of MSCs.

## Introduction

Bone is an active organ that undergoes persistent metabolism^1^. Maintaining the body’s bone mass balance depends on the steady state of bone tissue, which is regulated by osteoclasts and osteoblasts^2^. Mesenchymal stem cells (MSCs) are pluripotent stem cells that exist in connective tissues and are an important source of osteoblasts in the body^3^. Due to their functional properties, MSCs are considered to be promising cell types for various applications and are used most frequently in regenerative medicine, especially for bone repair^4–6^. However, the regulatory mechanism controlling the osteogenic differentiation of MSCs remains largely unknown and hampers the further application of MSC-based cell therapies^7^. Understanding the molecular mechanism that regulates the osteogenic differentiation of MSCs will therefore allow us to further accelerate the differentiation of MSCs to osteoblasts, shorten the treatment time, and improve the safety and effectiveness of the clinical treatment, which is of great significance for the clinical application of treatments.

As a genetic medium in the process of transcription and translation, RNA is thought to be only an intermediate product that transfers genetic information from DNA to protein. In recent years, research has found that there are also abundant epigenetic modifications on RNA, playing a critical role in modulating biological processes. *N*^6^-methyladenosine (m^6^A) is the most common RNA methylation modification in humans^8,9^. Existing research shows that under the control of methyltransferases and demethylases, m^6^A can change dynamically in different biological processes; in addition, m^6^A-binding proteins recognize m^6^A and are responsible for regulating the metabolism of RNA^10^. Studies have shown that m^6^A can regulate cell differentiation and participate in various pathophysiological processes. After downregulation of fat mass and obesity-associated protein (FTO) in adipose precursor cells, the adipogenic differentiation ability of the cells is weakened^11^; methyltransferase 14 (METTL14) participates in the self-renewal and proliferation of embryonic neural stem cells by regulating histone modifications^12^. Due to the strong correlation between m^6^A and cell differentiation, we wondered whether m^6^A is also involved in regulating the osteogenic differentiation of MSCs, which has not been fully elucidated.

The current study only found two demethylases, alkB homolog 5 (ALKBH5) and FTO. A previous study found that FTO plays a role in bone mass^13^. However, whether ALKBH5 can regulate the osteogenic differentiation of MSCs and its role are unclear. In our study, we demonstrated that conditional knockout of *Alkbh5* in bone marrow MSCs strengthened bone mass in mice. Mechanistically, we revealed that the m^6^A demethylase ALKBH5 plays a negative role in regulating the osteogenic capacity of MSCs by increasing the mRNA decay rate of protein arginine methyltransferase 6 (*PRMT6*) and discovered the phosphatidylinositol 3-kinase (PI3K)/AKT pathway as a crucial downstream target of the ALKBH5-PRMT6 axis. Together, our research unveils a different facet of the novel ALKBH5-PRMT6 axis that modulates the osteogenic differentiation of MSCs, which suggests a potential strategy for improving the efficiency of MSC-based tissue engineering for bone regeneration.

## Results

### Conditional knockout of *Alkbh5* expression in mouse MSCs strengthened bone mass

**Fig. 1:**
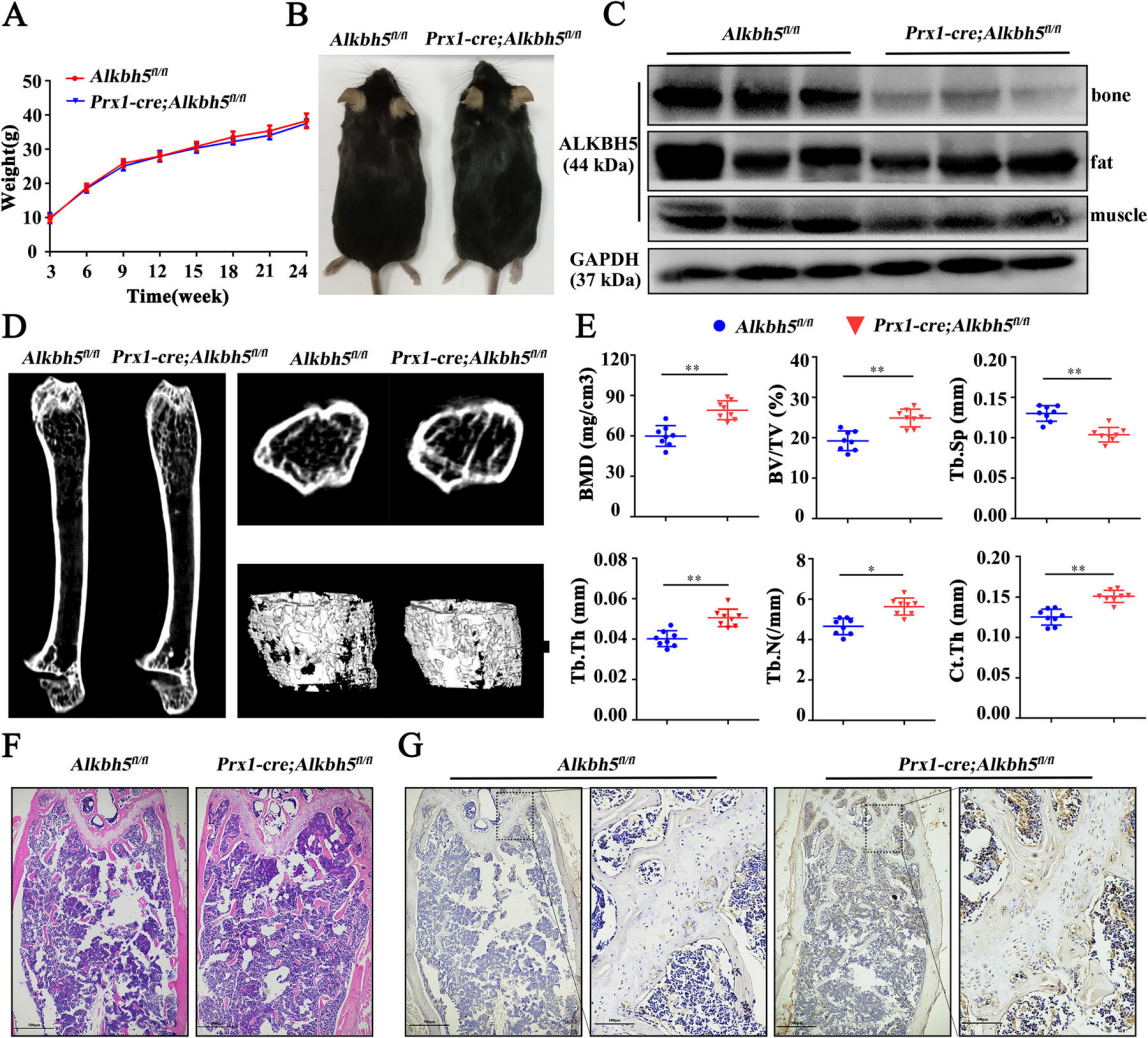
Conditional knockout of *Alkbh5* expression in mouse MSCs strengthened bone mass. **A** Growth curves for *Prx1-Cre; Alkbh5*^*fl/fl*^ and control mice over 24 wk (*n* = 8). **B** Representative photograph of a *Prx1-Cre; Alkbh5*^*fl/fl*^ mouse and an *Alkbh5*^*fl/fl*^ control littermate at 24 wk of age. **C** Western blotting to detect the expression of Alkbh5 in mouse femur, fat, and muscle. *n* = 8 in each group and only the results for three mice are displayed. **D** Representative computer renderings of bone structure in the femurs from *Prx1-Cre; Alkbh5*^*fl/fl*^ and *Alkbh5*^*fl/fl*^ mice at 24 wk (male, *n* = 8). **E** Quantitative μCT analyses of the distal end of femurs at 24 wk (male, *n* = 8). **F** Representative H&E staining of femoral sections from *Prx1-Cre; Alkbh5*^*fl/fl*^ and *Alkbh5*^*fl/fl*^ male mice. Scale bar, 100 µm (male, *n* = 8). **G** Representative Bglap immunohistochemical staining of femoral sections from *Prx1-Cre; Alkbh5*^*fl/fl*^ and *Alkbh5*^*fl/fl*^ male mice. Scale bars: left, 100 µm; right, 500 µm. All data are presented as the means ± SDs. **p < 0.01.

To study the role of Alkbh5 in the osteogenic differentiation of bone marrow MSCs, we first constructed *Prx1-Cre; Alkbh5*^*fl/fl*^ mice (Supplementary Fig. 1A, B). Compared with the *Alkbh5*^*fl/fl*^ control littermates, *Prx1-Cre; Alkbh5*^*fl/fl*^ mice showed no significant difference in size and weight (Fig. 1A, B). Western blotting detection confirmed that *Prx1-Cre; Alkbh5*^*fl/fl*^ mouse bone tissue completely lacks Alkbh5 expression, but no change in the expression of the Alkbh5 protein was observed in adipose tissue and muscle tissue (Fig. 1C).

Micro-computed tomography (µCT) was used to scan the femurs of the mice and the results showed that *Prx1-Cre; Alkbh5*^*fl/fl*^ mice had increased bone mass compared with their *Alkbh5*^*fl/fl*^ control littermates (Fig. 1D). Analysis of the trabecular bone of the distal femur metaphysis revealed that compared with those in *Alkbh5*^*fl/fl*^ mice, the bone mineral density and bone volume/tissue volume ratio (BV/TV) in *Prx1-Cre; Alkbh5*^*fl/fl*^ mice increased by 32% and 29%, respectively. Furthermore, *Alkbh5* deletion also increased the trabecular number (Tb. N), trabecular thickness (Tb. Th), and cortical thickness but decreased the trabecular separation (Fig. 1E). The hematoxylin and eosin (H&E) staining results were consistent with the µCT analysis results. *Prx1-Cre; Alkbh5*^*fl/fl*^ mice had more trabecular bones than their *Alkbh5*^*fl/fl*^ littermates (Fig. 1F). Immunohistochemical staining showed that the expression of bone gamma carboxyglutamate protein (Bglap) in bone tissue of *Prx1-Cre; Alkbh5*^*fl/fl*^ mice increased compared with that in their control littermates (Fig. 1G). Overall, the results indicate that conditional knockout of *Alkbh5* in mouse MSCs strengthened bone mass in mice.

### The overall level of m^6^A is upregulated during the osteogenic differentiation of MSCs

**Fig. 2:**
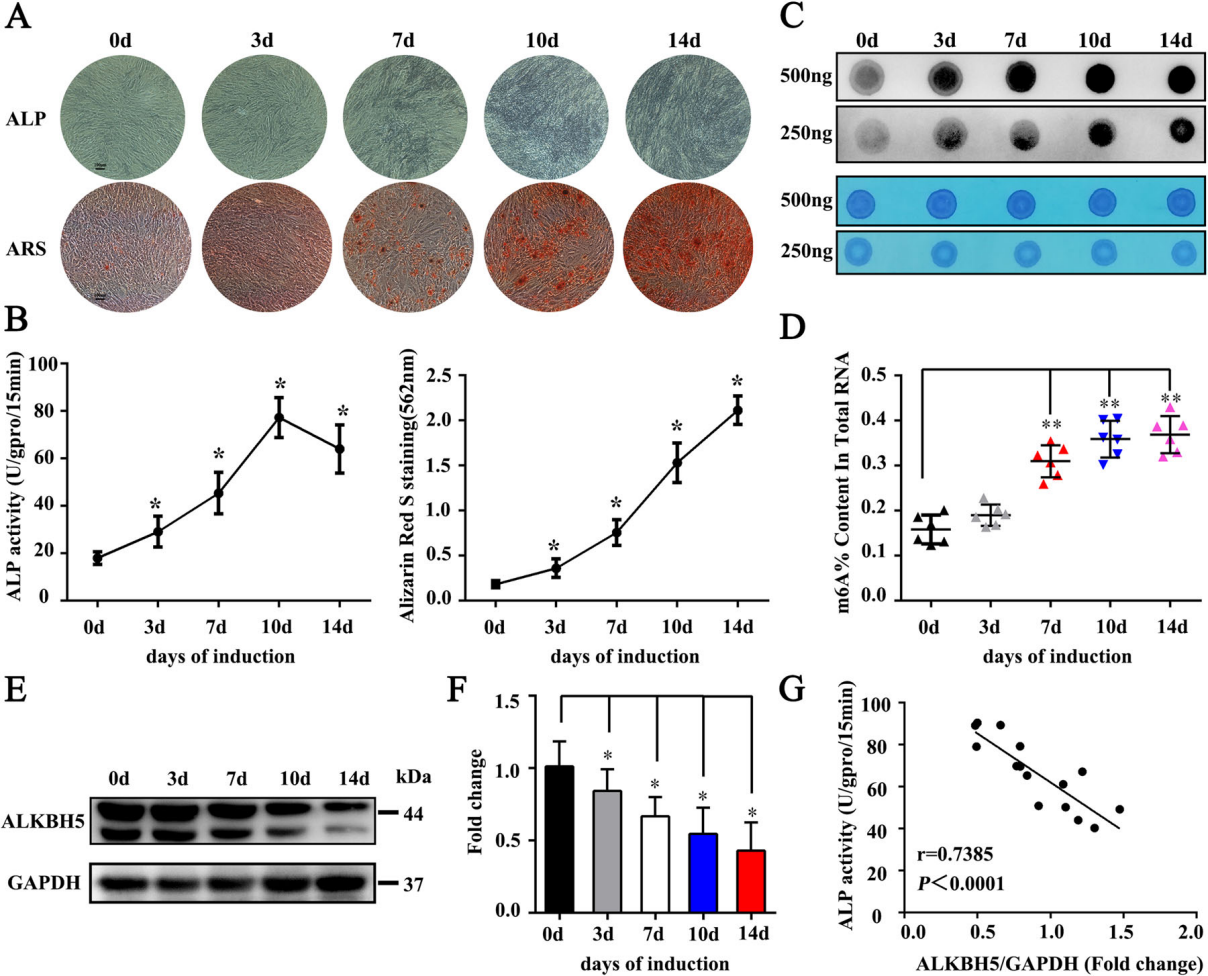
The overall m^6^A modification level increased during the osteogenic differentiation of MSCs. MSCs were cultured in osteogenic medium for up to 14 days. **A** ARS staining (top) and ALP staining (bottom) during the osteogenic differentiation of MSCs. Scale bar, 100 µm. **B** ALP activity was determined as units per gram of protein per 15 min and ARS staining was quantified as the absorbance at 562 nm (*n* = 3 independent experiments with three different MSC lines). **C** m^6^A dot blot detection of the overall m^6^A modification level at different osteogenic differentiation time points (*n* = 6). **D** An m^6^A RNA methylation assay was used to detect the overall m^6^A modification level at different time points (*n* = 6). **E**, **F** Protein level of ALKBH5 during osteogenic differentiation of MSCs (*n* = 9). **G** Correlation analysis of the expression level of ALKBH5 and ALP activity (*n* = 15). All data are presented as the means ± SDs. *p < 0.05.

We next considered whether ALKBH5 also plays an important role in the osteogenic differentiation of human MSCs. By selecting different induction time points for alizarin red S (ARS) staining, alkaline phosphatase (ALP) staining, and ALP activity detection, we confirmed the osteogenic differentiation ability of isolated and cultured MSCs in vitro (Fig. 2A, B), which was consistent with previous tests^14^. Next, we detected m^6^A methylation by an m^6^A mRNA dot blot and an m^6^A RNA methylation assay. As a result, the overall level of m^6^A gradually increased during osteogenic differentiation and peaked on the 14th day (Fig. 2C, D). Furthermore, the western blotting results showed that the expression of ALKBH5 gradually decreased (Fig. 2E, F). Correlation analysis showed that the expression level of ALKBH5 was negatively correlated with ALP activity (Fig. 2G). The above results suggest that the overall level of m^6^A during the osteogenic differentiation of MSCs was increased by the downregulation of the demethylating enzyme ALKBH5.

### ALKBH5 negatively regulates the osteogenic differentiation capacity of MSCs

**Fig. 3:**
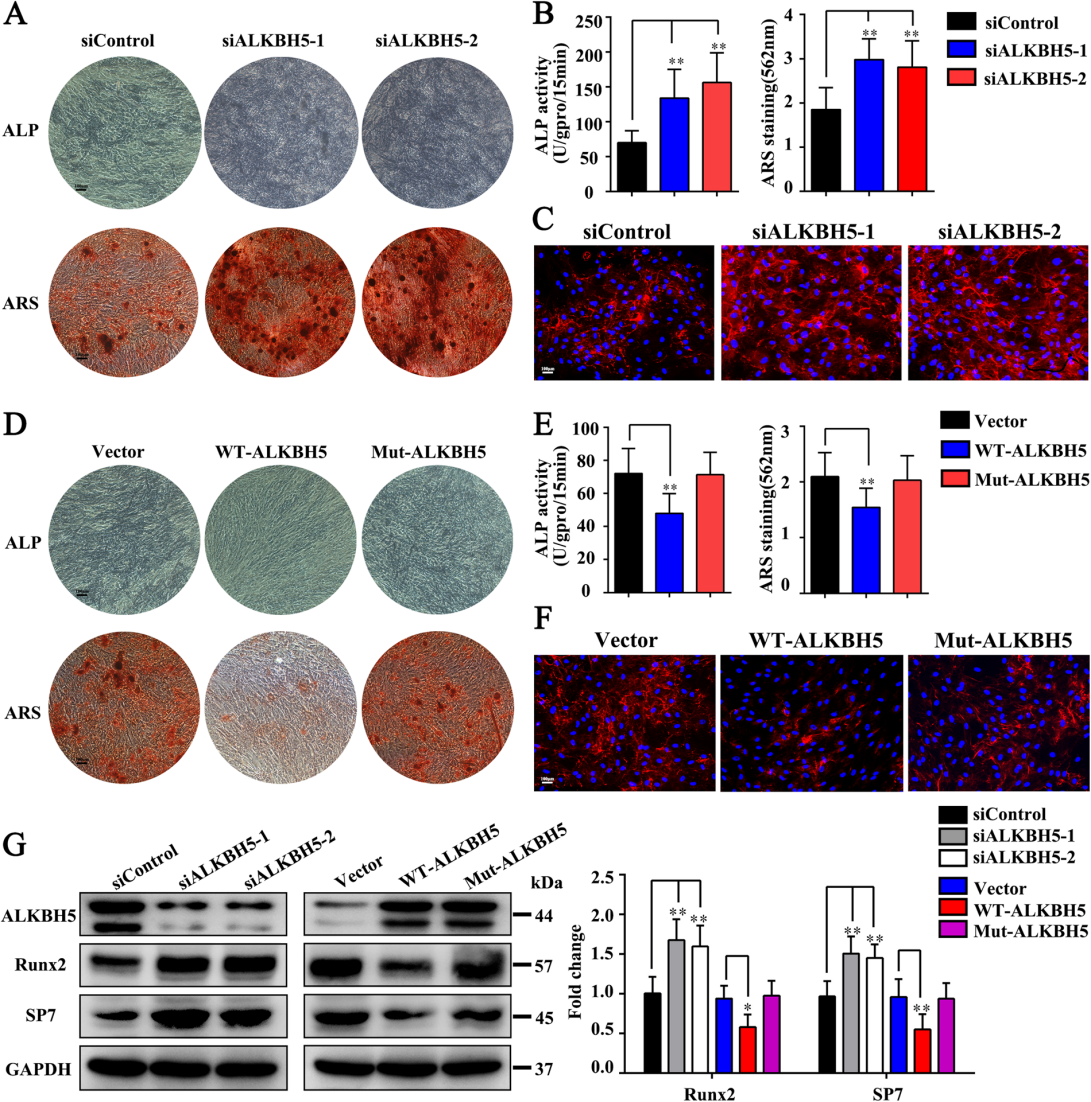
The m^6^A demethylase ALKBH5 negatively regulated the osteogenic differentiation of MSCs in vitro. ALKBH5 was knocked down and overexpressed using siRNA and lentivirus, respectively. **A** ALP staining and ARS staining in the ALKBH5 knockdown or control group. Scale bar, 100 µm. **B** ALP activity and ARS staining quantification in the ALKBH5 knockdown or control group (*n* = 3 independent experiments with three different MSC lines). **C** The immunofluorescence of collagen I in the ALKBH5 knockdown or control group. Scale bar, 100 µm (*n* = 3 independent experiments with three different MSC lines). **D** ALP staining and ARS staining after using wild-type lentivirus (WT-ALKBH5) and mutant lentivirus (Mut-ALKBH5) to overexpress ALKBH5. Scale bar, 100 µm. **E** ALP activity and ARS staining quantification in the ALKBH5 overexpression or control group (*n* = 3 independent experiments with three different MSC lines). **F** The immunofluorescence of collagen I in the ALKBH5 overexpression or control group. Scale bar, 100 µm (*n* = 3 independent experiments with three different MSC lines). **G** Protein levels of the osteogenesis-associated markers Runx2 and SP7 were determined by western blot analysis (*n* = 3 independent experiments with three different MSC lines). All data are presented as the means ± SDs. *p < 0.05.

Next, we detected the regulatory effect of ALKBH5 on osteogenic differentiation of MSCs. Quantitative PCR (qPCR) and western blotting verified that siALKBH5 had good knockout efficiency (Fig. 3G). The results showed that after interfering with ALKBH5 expression, ALP staining and activity increased. ARS staining found that the siALKBH5 group had a significantly greater number of calcium nodules than the control group (Fig. 3A, B). The immunofluorescence of collagen I showed increased expression in the siALKBH5 group compared to the control group (Fig. 3C). After overexpressing WT-ALKBH5, ALP staining and activity decreased. The ARS staining results were consistent with the ALP staining results (Fig. 3D, E). Studies have shown that a mutant ALKBH5 protein with the iron ligand residue H204 substituted to Ala completely lost its demethylation activity^15^. To determine whether the ALKBH5-mediated regulation of the osteogenic differentiation of MSCs is dependent on m^6^A modification, we also constructed the ALKBH5 catalytically active mutant Mut-ALKBH5 (H204A). The results showed that compared with the control group, there was no significant change in ALP staining, ALP activity, or ARS staining in the Mut-ALKBH5 group (Fig. 3D, E). The immunofluorescence of collagen I was generally consistent with the ARS staining results (Fig. 3F).

In addition, we determined the expression levels of the osteogenic markers RUNX family transcription factor 2 (RUNX2) and Sp7 transcription factor (SP7). The results showed that knocking down ALKBH5 increased the expression of RUNX2 and SP7, whereas overexpressing WT-ALKBH5 inhibited the expression of RUNX2 and SP7. However, overexpressing Mut-ALKBH5 did not change the expression of these two marker proteins (Fig. 3G). In summary, our data revealed that the demethylase ALKBH5 has a negative regulatory effect on the osteogenesis of MSCs through its m^6^A demethylation active site.

### Whole-transcriptome m^6^A-seq and RNA-seq detection of ALKBH5 downstream regulatory genes

**Fig. 4:**
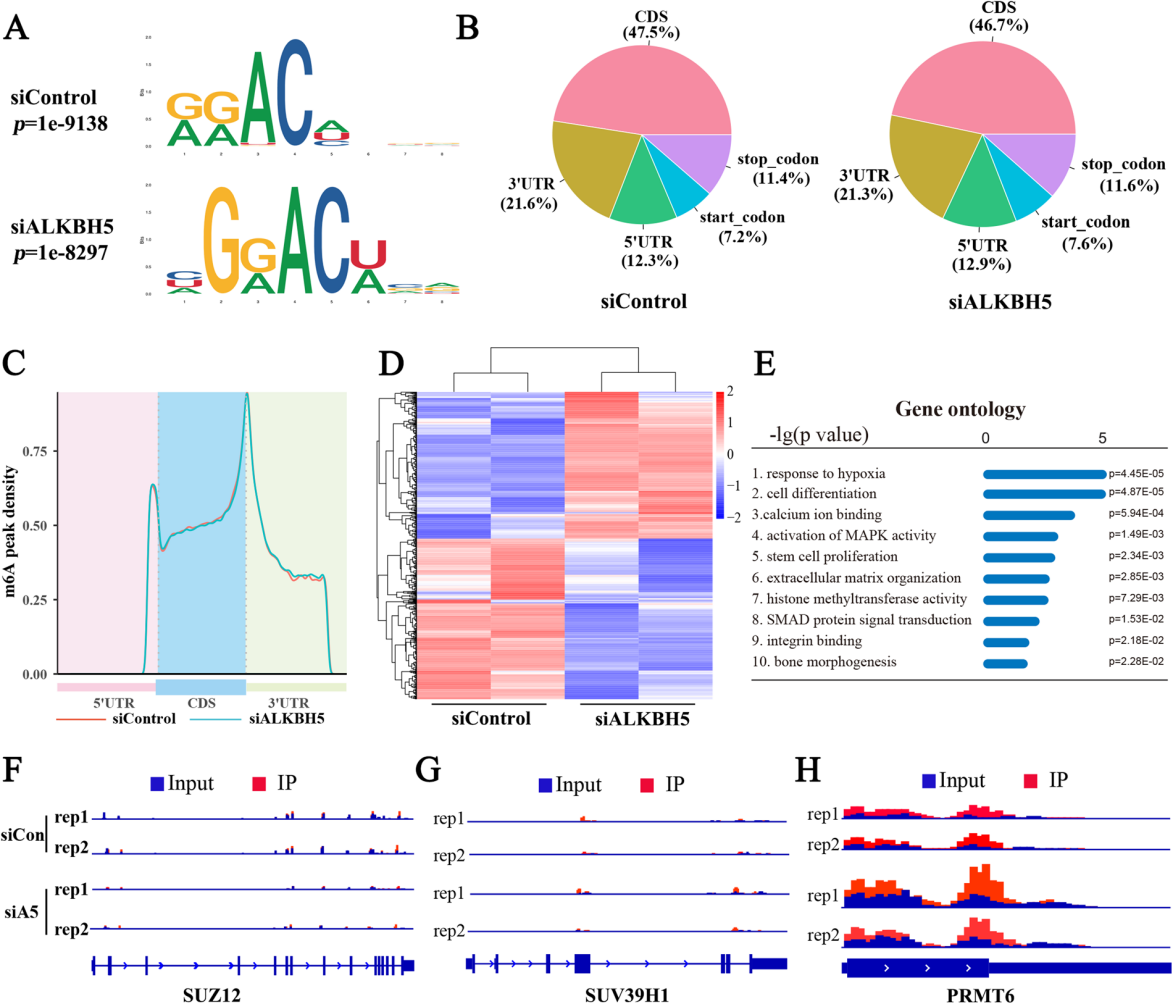
Whole-transcriptome m^6^A-seq and RNA-seq detection of ALKBH5 downstream regulatory genes. **A** Specific motif sequences of the siControl and siALKBH5 groups (two biological repeats per group). **B** Proportion of m^6^A peak distribution in the 5′-UTR, start codon, CDS, stop codon or 3′-UTR across the entire set of mRNA transcripts. **C** Density distribution of m^6^A peaks across mRNA transcripts. **D** Heatmap of genes with significantly different expression between the siControl and siALKBH5 groups. siCon-1 and siCon-2 are biological repeats; **E** GO enrichment analysis of differentially expressed genes. **F**–**H** IGV software analysis of the m^6^A peaks of *SUZ12*, *SUV39H1*, and *PRMT6*.

As ALKBH5 regulated the osteogenic differentiation of MSCs through m^6^A demethylation activity, we performed m^6^A sequencing (m^6^A-seq) on the control group and siALKBH5 group with two independent biological replicates. Consistent with previous m^6^A-seq results^16,17^, the canonical RRACH motif was highly enriched within m^6^A sites in both the control and small interfering RNA (siRNA) groups (Fig. 4A). These m^6^A modifications were predominantly located in the coding sequence and the 3′-untranslated region (Fig. 4B). m^6^A-seq analysis identified 43,872 and 44,701 m^6^A peaks from 14,472 to 14,637 m^6^A-modified genes in the control and siALKBH5 groups, respectively (Supplementary Fig. 2A). We further analyzed the total m^6^A peak density of mRNAs according to the m^6^A-seq results. The analysis showed that m^6^A peaks were abundant in the vicinity of the start and stop codons, especially at the position of the stop codon (Fig. 4C).

Next, we used RNA sequencing (RNA-seq) to reveal gene expression changes resulting from decreased ALKBH5 expression in MSCs. RNA-seq results showed that after knocking down ALKBH5 expression, there were a total of 790 genes with expression changes, including 377 upregulated genes and 413 downregulated genes (Fig. 4D and Supplementary Fig. 2B). Gene Ontology (GO) enrichment analysis of these changed genes indicated that a handful of the genes were associated with cell differentiation, histone methyltransferase activity, and activation of mitogen-activated protein kinase activity (Fig. 4E), and Kyoto Encyclopedia of Genes and Genomes (KEGG) pathway enrichment analyses also revealed that genes altered by ALKBH5 were clustered in signaling pathways regulating the pluripotency of stem cells, Extracellular matrix (ECM)–receptor interactions, and the PI3K/Akt signaling pathway (Supplementary Fig. 2C), further indicating that ALKBH5 plays a role in the regulation of osteogenic differentiation of MSCs. By comparing the peak calling of related genes in the histone methyltransferase activity pathway through Integrative Genomics Viewer (IGV) analysis, it was found that *SUV39H1* and *SUZ12* did not have specific m^6^A peaks (Fig. 4F, G), whereas the *PRMT6* was modified by m^6^A and the m^6^A peak in the siALKBH5 group was higher than that in the control group (Fig. 4H). Therefore, we speculated that PRMT6 may be the downstream target of ALKBH5 and selected this protein for further analysis.

### ALKBH5 downregulates the expression of PRMT6 through m^6^A modification

**Fig. 5:**
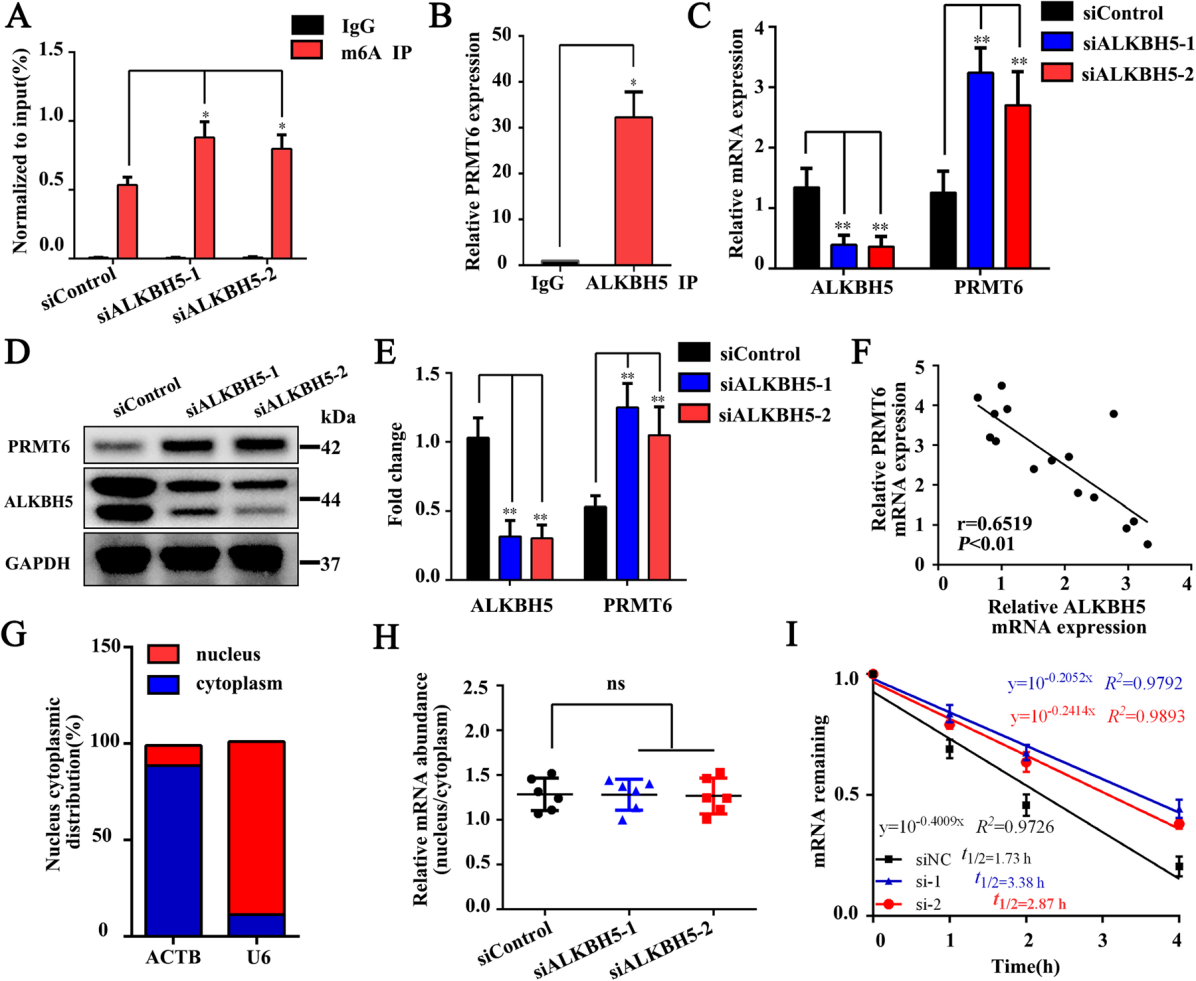
ALKBH5 downregulates the expression of PRMT6 through m^6^A modification. **A** meRIP-qPCR analysis of *PRMT6* mRNA in the siControl and siALKBH5 groups (*n* = 6). **B** RIP-qPCR detected whether ALKBH5 and *PRMT6* were directly bound (*n* = 6). **C** qPCR results of *PRMT6* after interfering with ALKBH5 expression (*n* = 9). **D**, **E** After interfering with ALKBH5 expression, western blotting was used to detect and quantify PRMT6 (*n* = 9). **F** Correlation analysis of *PRMT6* and *ALKBH5* during osteogenic differentiation of MSCs (*n* = 15). **G** Nuclear and cytoplasmic fractionation assays following qPCR in the siControl and siALKBH5 groups (*n* = 6). actin β (*ACTB*) and U6 small nuclear 1 (*U6)* were used as the positive controls. **H** After diminishing ALKBH5 expression, qPCR was used to detect the distribution of *PRMT6* in the cytoplasm and nucleus (*n* = 6). **I** The RNA lifetime for *PRMT6* in MSCs transfected with control siRNA or siRNA targeting ALKBH5 was determined by monitoring transcript abundance after adding actinomycin D (*n* = 9). All data are presented as the means ± SDs. **P < 0.01; ns, not statistically significant.

To determine whether PRMT6 is directly regulated by ALKBH5, we found through methylated RNA immunoprecipitation qPCR (meRIP-qPCR) experiments that compared with the IgG group, m^6^A-specific antibodies can significantly enrich *PRMT6*, which is consistent with the m^6^A-seq results; after knocking down ALKBH5 expression, the enrichment of m^6^A methylation in *PRMT6* was higher than that in the siControl group (Fig. 5A). Furthermore, the results of RNA immunoprecipitation qPCR (RIP-qPCR) showed that compared with the IgG group, the anti-ALKBH5 antibody significantly enriched *PRMT6* (Fig. 5B). We found by qPCR and western blotting that knocking down ALKBH5 increased the expression of PRMT6 (Fig. 5C–E). The correlation analysis showed that there was a negative correlation between *PRMT6* and *ALKBH5* expression (Fig. 5F). The results confirmed that the methylation level of *PRMT6* was regulated by ALKBH5.

Next, we explored the specific regulatory mechanism of ALKBH5-mediated downregulation of PRMT6. qPCR results showed successful separation of cytoplasmic and nuclear mRNA (Fig. 5G). However, comparison of the expression of *PRMT6* in the cytoplasm and nucleus showed that there was no difference between the control group and siALKBH5 group (Fig. 5H). Subsequently, we detected the mRNA decay rate of *PRMT6* after knocking down ALKBH5 expression. The results showed that the degradation rate of *PRMT6* was significantly slowed in the siALKBH5 group (Fig. 5I), indicating that ALKBH5 regulates the expression of PRMT6 mainly by affecting the mRNA degradation rate.

### ALKBH5 regulates the osteogenic differentiation ability of MSCs mainly through PRMT6

**Fig. 6:**
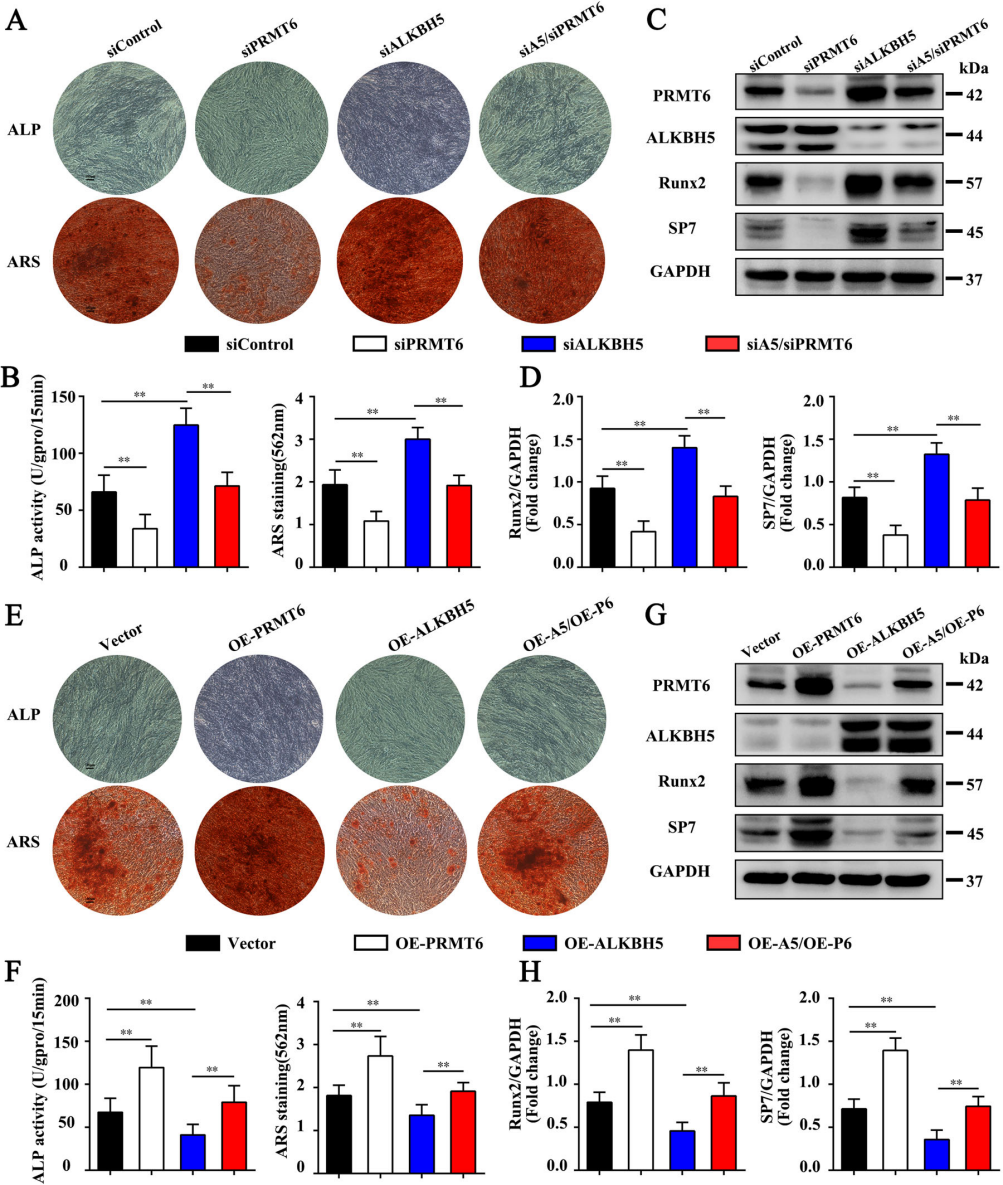
ALKBH5 regulates the osteogenic differentiation ability of MSCs through PRMT6. **A** ALP staining and ARS staining after simultaneous interference with ALKBH5 and PRMT6 expression. Scale bar, 100 µm. **B** ALP activity and ARS staining quantification after simultaneous interference with ALKBH5 and PRMT6 expression (*n* = 3 independent experiments with three different MSC lines). **C**, **D** Protein levels of the osteogenesis-associated markers RUNX2 and SP7 were determined by western blot analysis (*n* = 3 independent experiments with three different MSC lines). **E** ALP staining and ARS staining after overexpression of ALKBH5 and PRMT6. Scale bar, 100 µm. **F** ALP activity and ARS staining quantification after overexpression of ALKBH5 and PRMT6 (*n* = 3 independent experiments with three different MSC lines). **G**, **H** Western blot detection of RUNX2 and SP7 expression after overexpression of ALKBH5 and PRMT6 (*n* = 3 independent experiments with three different MSC lines). All data are presented as the means ± SDs. **p < 0.01.

As the functions of PRMT6 in the osteogenic differentiation of MSCs remain unclear, we first examined the expression pattern of PRMT6 during the osteogenic differentiation of MSCs. The results demonstrated that RPMT6 expression was upregulated after induction to the osteogenic lineage (Supplementary Fig. 3A). The correlation analysis showed that there was a positive correlation between *PRMT6* and *RUNX2* or *SP7* mRNA expression (Supplementary Fig. 3B). ALP staining and activity were markedly suppressed upon PRMT6 knockdown. ARS staining showed that calcium nodules were significantly reduced after PRMT6 knockdown (Fig. 6A, B). Western blotting results showed that the expression of RUNX2 and SP7 decreased in the siPRMT6 group (Fig. 6C, D). In contrast, the ALP assay values and ARS staining were both increased after overexpressing PRMT6 (Fig. 6E, F). Western blotting demonstrated that PRMT6 overexpression increased the expression of RUNX2 and SP7 (Fig. 6G, H). Our data indicate that PRMT6 positively regulates the osteogenic differentiation of MSCs.

Further studies demonstrated that simultaneous knockdown of ALKBH5 and PRMT6 decreased ARS and ALP assay values to the levels observed in the siControl group compared with the siALKBH5 group (Fig. 6A, B). In addition, simultaneous knockdown of ALKBH5 and PRMT6 reduced the expression of RUNX2 and SP7 to the level in the control group (Fig. 6C, D). In contrast, simultaneous overexpression of ALKBH5 and PRMT6 rescued the ARS and ALP results compared with those in the OE-ALKBH5 group (Fig. 6E, F). Western blotting results showed that overexpression of ALKBH5 and PRMT6 simultaneously increased the expression of RUNX2 and SP7 compared with that in the OE-ALKBH5 group (Fig. 6G, H). These results suggest that PRMT6 is a critical downstream target of ALKBH5-mediated inhibition of osteogenic differentiation of MSCs.

### The ALKBH5-PRMT6 axis controls the activation of the AKT signaling pathway to modulate the osteogenesis of MSCs

**Fig. 7:**
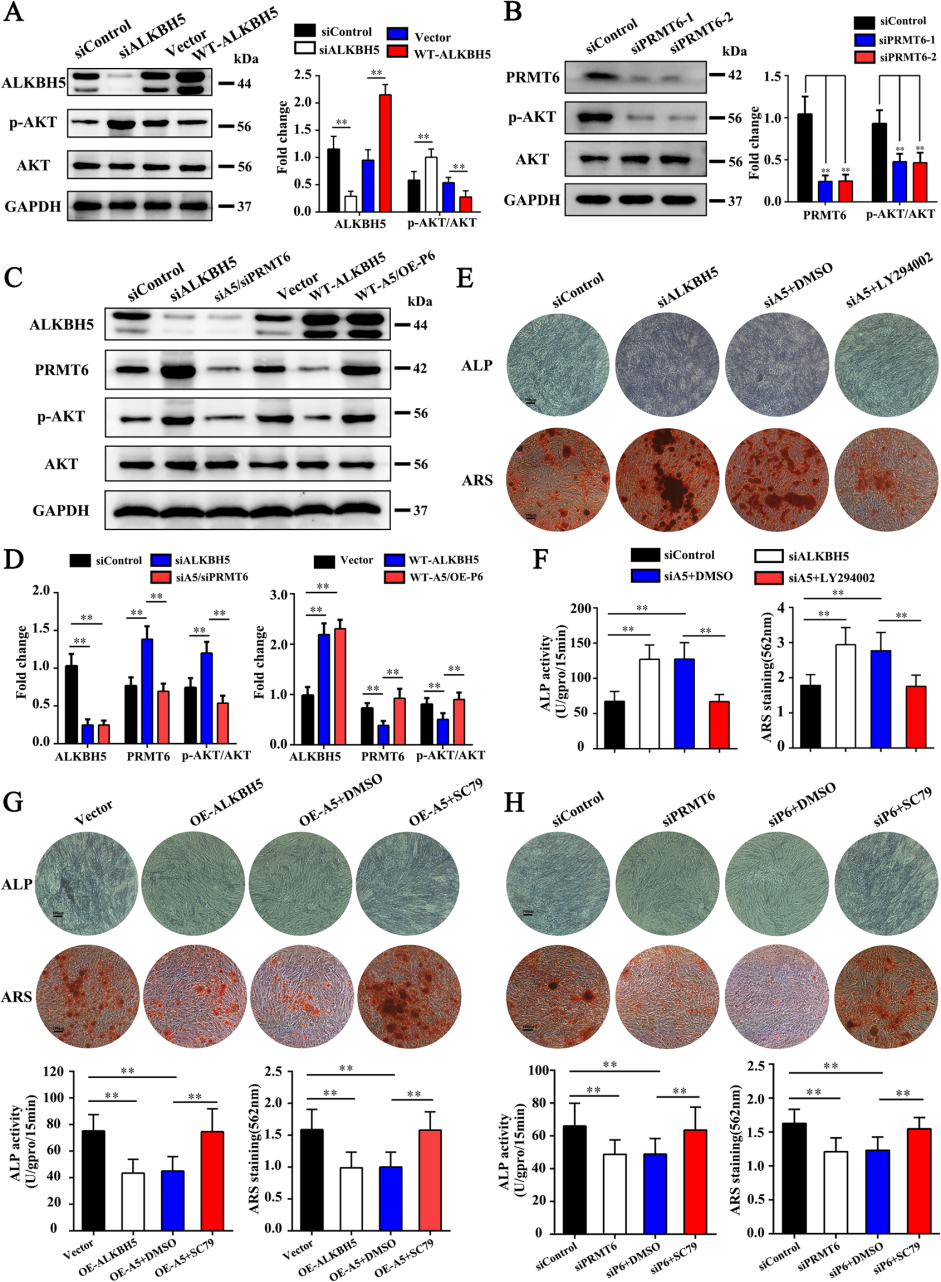
The ALKBH5-PRMT6 axis controls the activation of the AKT signaling pathway to modulate the osteogenesis of MSCs. **A** Western blot detection of AKT phosphorylation level after interference with ALKBH5 expression. **B** Western blot detection of AKT phosphorylation level after knocking down PRMT6 expression. **C**, **D** Western blot detection of AKT phosphorylation level after simultaneous knockdown of ALKBH5 and PRMT6, and simultaneous overexpression of ALKBH5 and PRMT6. **E**, **F** ALP assay and ARS assay after adding LY294002 (PI3K/AKT pathway inhibitor), while knocking down ALKBH5. Scale bar, 100 µm. **G** ALP assay and ARS assay after adding SC79 (PI3K/AKT pathway activator), while overexpressing ALKBH5. Scale bar, 100 µm. **H** ALP assay and ARS assay after adding SC79, while knocking down PRMT6. Scale bar, 100 µm. All data are presented as the means ± SDs. **p < 0.01 (*n* = 3 independent experiments with three different MSC lines).

Previous studies demonstrated that PRMT6 can regulate the activation of PI3K/AKT signaling pathway^18,19^ and the PI3K/AKT pathway plays an important role in regulating the osteogenesis of MSCs^20^. In addition, KEGG pathway enrichment analyses found that the gene-enriched pathway contains the PI3K/AKT pathway (Supplementary Fig. 1C). Therefore, we speculated that the ALKBH5-PRMT6 axis regulates osteogenesis of MSCs through the PI3K/AKT pathway. Western blotting results showed that the activation level of the AKT signaling pathway was increased after ALKBH5 knockdown but decreased in the WT-ALKBH5 group (Fig. 7A). In addition, the phosphorylation level of AKT was decreased in the siPRMT6 group (Fig. 7B). Further studies demonstrated that simultaneous knockdown of ALKBH5 and PRMT6 decreased the activation level of AKT to those observed in the siControl group compared with the siALKBH5 group, whereas simultaneous overexpression of ALKBH5 and PRMT6 rescued the phosphorylation level of AKT compared with that in the OE-ALKBH5 group (Fig. 7C, D).

Subsequently, we evaluated the role of the PI3K/AKT pathway in the osteogenic differentiation of MSCs regulated by the ALKBH5-PRMT6 axis. Adding LY294002 (PI3K/AKT pathway inhibitor) while knocking down ALKBH5 decreased ARS and ALP assays to the level observed in the siControl group compared with siALKBH5 group (Fig. 7E, F), whereas adding SC79 (PI3K/AKT pathway activator) while overexpressing ALKBH5 rescued ARS and ALP assays compared with that in the OE-ALKBH5 group (Fig. 7G). Western blotting results demonstrated that the expression of RUNX2 and SP7 decreased in the siALKBH5 + LY294002 group compared with the siALKBH5 group (Supplementary Fig. 4A) but increased in the WT-ALKBH5 + SC79 group compared with the WT-ALKBH5 group (Supplementary Fig. 4B). Furthermore, ARS and ALP assays were increased to the level observed in the siControl group compared with the siPRMT6 group after adding SC79, while knocking down PRMT6 (Fig. 7H). Western blotting results showed that the expression of RUNX2 and SP7 in the siPRMT6 + SC79 group was rescued to the level of the siControl group (Supplementary Fig. 4C).

**Fig. 8:**
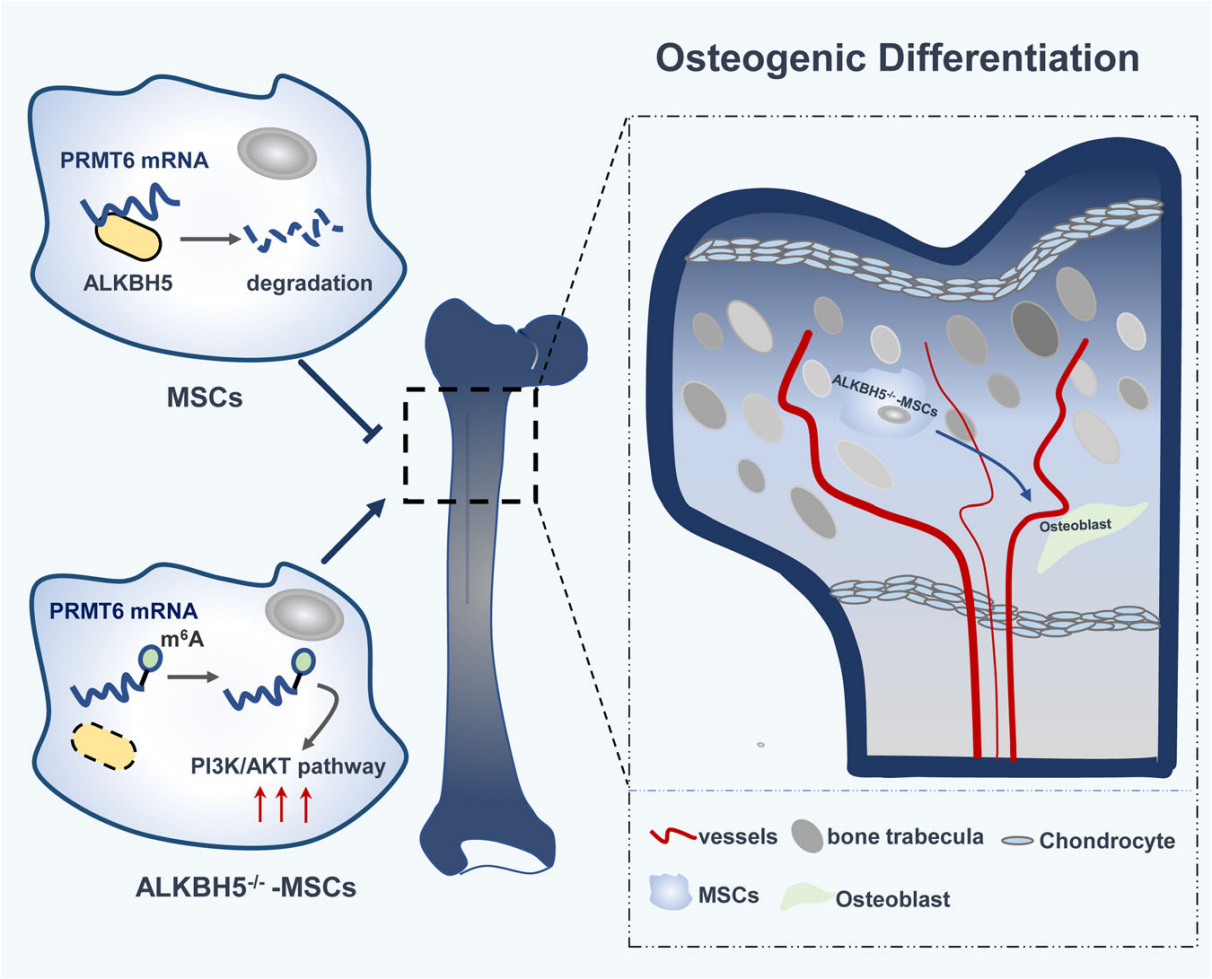
Working model depicting how ALKBH5 regulates the osteogenic differentiation of MSCs. ALKBH5 negatively regulates the osteogenic differentiation of MSCs by increasing the mRNA decay rate of *PRMT6*, so as to inhibit the activation of PI3K/AKT pathway in bone development.

## Discussion

In this study, we demonstrated that conditional knockout of *Alkbh5* in bone marrow MSCs strengthened bone mass in mice. The overall level of m^6^A modification increased during the osteogenic differentiation of MSCs and the expression of the demethylase ALKBH5 gradually decreased, which negatively regulated the osteogenic differentiation of MSCs. Further research found that ALKBH5 accelerates the degradation rate of PRMT6 in an m^6^A-dependent manner, and that PRMT6 influences the activity of the PI3K/AKT signaling pathway to regulate the osteogenic differentiation of MSCs in vitro (Fig. 8).

The discovery of the m^6^A modification expands our understanding of epigenetic regulation in the life sciences. To date, a number of studies have shown that m^6^A modification participates in a variety of biological functions and plays an important role in regulating cell differentiation^21^. Interfering with the expression of METTL3 in pluripotent embryonic stem cells affects the differentiation of embryonic stem cells and makes them stay in a naive state^22^; imbalance of m^6^A modification regulation impairs the differentiation of fat precursor cells^11^ and hematopoietic stem cells^23^. In our study, we found that the overall level of m^6^A modification during osteogenesis of MSCs increased, demonstrating that m^6^A modification has different levels of activity before and after osteogenic differentiation. Subsequently, correlation analysis showed that the expression level of ALKBH5 was negatively correlated with ALP activity. Through functional studies, our research demonstrated that the demethylase ALKBH5 negatively regulates the osteogenic differentiation of MSCs through m^6^A modification, which has not been directly reported before.

To uncover the downstream target of ALKBH5, we performed m^6^A-seq and RNA-seq. By performing GO enrichment analysis, we found that a handful of genes were associated with histone methyltransferase activity. Previous studies have found that m^6^A modification can play a regulatory role through the histone pathway^12^ and histone modification plays an important role in regulating the osteogenic differentiation of MSCs^24,25^. Therefore, we wondered whether ALKBH5 can regulate the osteogenic differentiation of MSCs through the histone pathway. Then, we found that PRMT6 was a critical downstream target of ALKBH5. PRMT6 can catalyze the asymmetric dimethylation of histone H3 (Arg2 and Arg42), histone H2A (Arg29), and nonhistone proteins, and regulate cell signal transduction, transcriptional regulation, RNA splicing, and DNA repair^26,27^. At a mechanistic level, we demonstrated that ALKBH5 regulates the expression of PRMT6 mainly by affecting the degradation rate of *PRMT6* mRNA but not by regulating its nucleocytoplasmic transport. We also found for the first time that PRMT6 promotes the osteogenic differentiation of MSCs. Furthermore, we uncovered that the PI3K/AKT pathway is an important downstream signaling pathway of the ALKBH5-PRMT6 axis. The PI3K/AKT signaling pathway plays not only an important role in regulating MSC osteogenic differentiation but also a key regulatory pathway for cell proliferation, growth, and survival^28^. Recent studies have indicated that m^6^A methylation regulates the activity of the PI3K/AKT pathway in endometrial cancers^29^ and acute myeloid leukemia^30^. Our research unexpectedly revealed that the demethylase ALKBH5 also influences the activity of the PI3K/AKT pathway in MSCs to regulate osteogenic differentiation. However, the specific genes regulated by the ALKBH5-PRMT6 axis in the PI3K/AKT pathway need to be further explored.

A primary study found that ALKBH5 is highly expressed in mouse testes and participates in the sperm development of mice^15^. A subsequent study found that ALKBH5 is essential for maintaining mouse brain development under hypobaric hypoxia^31^. In our research, we found that knocking out ALKBH5 in mouse MSCs had no significant effect on the growth of mice, but the bone mass and osteogenic activity of osteoblasts in *Prx1-Cre; Alkbh5*^*fl/fl*^ mice increased compared with that in their control littermates. Recent studies have reported that the conditional knockout mice *Prx1-Cre;Mettl3* ^*fl/fl*^ and *Lepr-Cre;Mettl3* ^*fl/fl*^ have lower bone mass than their control littermates, proving that METTL3 has a positive effect on regulating the osteogenic differentiation of MSCs^32^. In addition, two other studies have confirmed the above results^33,34^. By generating *Prx1-Cre; Alkbh5*^*fl/fl*^ mice, our data show that changing the m^6^A modification by regulating the expression of the methyltransferase METTL3 or demethylase ALKBH5 can lead to the regulation of MSC differentiation. Our research interestingly expands the understanding of the m^6^A modification-mediated regulation of the osteogenic differentiation of MSCs from a different aspect.

Under the influence of aging or pathological factors, the balance of osteoblasts and osteoclasts is often disrupted, resulting in a decrease in bone mass. Therefore, how to promote the conversion of bone tissue to osteoblasts and reverse the negative balance is of great significance for patients with low bone mass. Due to the critical role of m^6^A in modulating pathophysiological processes, some researchers are devoted to exploring small-molecule compounds related to methylases. Selberg et al.^35^ identified small-molecule ligands that increase m^6^A levels in RNA. In terms of inhibitors, the study also found several small-molecule inhibitors of the m^6^A demethylase FTO. Huang et al.^36^ developed a small-molecule compound, FB23-2, which can inhibit FTO, and found that FB23-2 significantly inhibits the progression of acute myeloid leukemia. Peng et al.^37^ identified entacapone, a U.S. Food and Drug Administration-approved drug that can serve as FTO inhibitors to treat metabolic disorders such as obesity and diabetes. A previous study also demonstrated that the nonsteroidal anti-inflammatory drug meclofenamic acid acts as a highly selective inhibitor of FTO^38^. Based on the studies reported above, we believe that the development of small-molecule compounds for methylase will have excellent application prospects in clinical treatment. Our research demonstrated that the demethylating enzyme ALKBH5 inhibits the osteogenic differentiation ability of MSCs. In future research, we can develop small-molecule inhibitors against ALKBH5, which will help improve the clinical effect of MSC transplantation in the treatment of low bone mass-related diseases.

In summary, we uncover the critical role of the novel ALKBH5-PRMT6 axis in modulating the osteogenic differentiation of MSCs. Our research expands the understanding of the role of m^6^A modification in regulating MSCs osteogenic differentiation, which is expected to provide new strategies for the treatment of patients with low bone mass by using MSCs. However, additional research still needs to be performed. To further confirm the therapeutic effect of ALKBH5, it is necessary to use pretreated MSCs to repair bone defects or osteoporosis and we are currently preparing to apply this theory to preclinical practice.

## Materials and methods

### Generation of conditional *Alkbh5*-knockout mice

*Alkbh5*^*fl/+*^ mice with C57BL/6 background were generated by Cyagen (Suzhou, China) using CRISPR/Cas-mediated genome engineering. Briefly, the *Alkbh5* gene (NCBI Reference Sequence: NM_172943, Ensembl: ENSMUSG00000042650) is located on mouse chromosome 11. Exon 1 was selected as the conditional knockout region. To engineer the targeting vector, homologous arms and the Conditional knockout (CKO) region were generated by PCR using the BAC clone RP23-329 M3 as the template. Cas9, guide RNA, and the targeting vector were coinjected into fertilized eggs for CKO mouse production. The pups were genotyped by PCR followed by sequencing analysis.

*Prx1-Cre* transgenic mice were purchased from The Jackson Laboratory. We crossed *Prx1-Cre* mice with *Alkbh5*^*fl/+*^ mice to obtain *Prx1-Cre; Alkbh5*^*fl/+*^ mice as heterozygous conditional *Alkbh5*-knockout mice. By mating *Prx1-Cre; Alkbh5* ^*fl/+*^ male mice with *Alkbh5*^*fl/fl*^ female mice, we obtained *Prx1-Cre; Alkbh5*^*fl/fl*^ mice as homozygous conditional Alkbh5-knockout mice. The genotype of the transgenic mice was identified by PCR analyses of genomic DNA extracted from mouse tails. Primers for floxed *Alkbh5*-knockout allele genotyping were as follows: forward (5′-CAGGTTTGAAGTGGCCATAGTAGC-3′) and reverse (5′-GAGGCCAAGACAGGAGAATCAGAC-3′). Primers for Cre transgene genotyping were as follows: forward (5′-GCTCTGATGTTGGCAAAGGGGT-3′) and reverse (5′-AACATCTTCAGGTTCTGCGGG-3′). All mice were bred and maintained under specific pathogen-free conditions. All procedures involving animals were approved by the Animal Use and Care Committee of the Eighth Affiliated Hospital of Sun Yat-sen University.

### MSC isolation and expansion

This study was approved by the Ethics Committee of Sun Yat-sen Memorial Hospital of Sun Yat-sen University, Guangzhou, China. Recruited healthy volunteers aged 20–30 years were fully informed of the relevant precautions and potential risks of bone marrow extraction, and signed the informed consent form. Bone marrow was collected from the posterior superior iliac spine of the volunteers and the MSCs were separated by density gradient centrifugation. The MSCs were resuspended in Dulbecco’s modified Eagle’s medium (DMEM, 1000 mg/L glucose, Gibco) containing 10% fetal bovine serum (FBS, Hangzhou Sijiqing Biological Engineering Material Company, Limited) and placed in a cell incubator containing 5% CO_2_ at 37 °C. After 48 h, the medium was changed to remove nonadherent cells. After 3 days, the medium was changed once. When the cell density reached 80–90%, the cells were digested with trypsin containing 0.53 mM EDTA and re-seeded in new flasks at passage 1. Cells from passage 3 to passage 5 were used for experiments.

### Osteogenic differentiation induction

MSCs were seeded in a 12-well plate at a density of 0.6 × 10^5^/well. After 12 h, when the cells adhered to the wells, the culture medium of the MSCs was changed to osteogenic differentiation medium consisting of DMEM (1000 mg/L glucose) with 10% FBS, 100 IU/mL penicillin, 100 IU/mL streptomycin, 0.1 μM dexamethasone, 10 mM β-glycerol phosphate, and 50 μM ascorbic acid (Sigma-Aldrich). The medium was changed every 3 days and continued to be induced to the desired time point.

### ARS staining and quantification

The original medium was discarded from the well plate, 500 μl of 4% paraformaldehyde was added to each well and the plate was placed at room temperature for 30 min to fix the cells. After paraformaldehyde fixation, the plate was rinsed twice with phosphate-buffered saline (PBS), 500 μl of 1% ARS (pH 4.3) was added, and staining was performed for 20 min. Then, PBS was added to rinse the plate and the central field of view of the well plate was selected under the microscope to be photographed. For ARS quantification, 10% cetylpyridinium chloride monohydrate (Sigma-Aldrich) was used to destain the cells for 1 h at room temperature. Then, 200 μl of the liquid was transferred to a 96-well plate and the spectrophotometric absorbance was measured at 562 nm.

### ALP staining and activity assay

ALP staining was performed according to the instructions of the BCIP/NBT Alkaline Phosphatase Color Development Kit (Beyotime Institute of Biotechnology). Briefly, MSCs were fixed in 4% paraformaldehyde for 15 min and then stained with mixture solution at 37 °C in the dark for 15 min. The central field of view of the well plate was selected to be photographed under the microscope.

For the ALP activity assay, an ALP assay kit (Nanjing Jiancheng Bioengineering Institute, China) was used. Then, 80 μl of radioimmunoprecipitation assay (RIPA) buffer was added to lyse cells in a well plate, followed by centrifugation at 14,000 r.p.m. for 10 min. A 96-well plate was prepared and 50 μl of A/1 solution and 50 μl of B/2 solution were added. Then, 30 μl of deionized water was added to the blank well, 30 μl of control solution was added to the standard well, and 30 μl of sample supernatant was added to the sample well. Then, the 96-well plate was placed in a 37 °C incubator for 15 min. After adding stop solution, the absorbance was measured at 405 nm. In addition, the protein concentration of the sample was determined by the Bicinchoninic acid (BCA) method, and finally, the ALP activity was calculated based on the measured ALP absorbance (optical density, OD) value and protein concentration; the unit of ALP activity was unit/g pro/15 min.

### Western blotting

RIPA solution containing phosphatase inhibitor and protease inhibitor was added to lyse the cells. After measuring the protein concentration by the BCA method, equal amounts of each sample were diluted in 5× sodium dodecyl sulfate (SDS) loading buffer and denatured by boiling. Protein lysates were separated by SDS-polyacrylamide gel electrophoresis and transferred to polyvinylidene difluoride membranes (Millipore). The membranes were blocked in 5% nonfat dry milk dissolved in TBST (150 mM NaCl, 50 mM Tris-HCl pH 7.5, and 0.05% Tween-20) at room temperature for 1 h and then incubated overnight with primary antibodies against ALKBH5 (1 : 1000, ab195377, Abcam), total AKT (1/5000, ab179463, Abcam), p-AKT (1 : 1000, ab38449, Abcam), SP7 (1 : 1000, ab209484, Abcam), RUNX2 (1 : 1000, ab23981, Abcam), PRMT6 (1 : 1000, 14641 S, Cell Signaling Technology), and glyceraldehyde 3-phosphate dehydrogenase (GAPDH; 1 : 3000, AF0006, Beyotime) overnight at 4 °C. The protein signals were detected using chemiluminescent reagents (Millipore) according to the manufacturer’s instructions.

### Quantitative reverse-transcriptase PCR

Total RNA from MSCs was extracted using TRIzol (Invitrogen) reagent. The RNA concentration was measured with a NanoDrop 2000 (Thermo Fisher Scientific). cDNA was transcribed by using PrimeScript RT Master Mix (Takara). Quantitative reverse-transcriptase PCR was performed using SYBR Green Premix Ex Taq (TaKaRa) in a Light-Cycler® 480 PCR System (Roche). Relative gene expression was normalized to GAPDH expression using the 2^−ΔΔCt^ method. The primers are listed in Supplementary Table 1.

### Cells transfection and chemical inhibition

The siRNAs were designed and synthesized by GenePharma (Shanghai, China). Three interference sequences were designed for the target gene. The interference efficiency was verified by qPCR and western blotting methods. Two sequences with a knockdown efficiency of >70% were selected for further experiments. Details of the sequences are shown in Supplementary Table 2. Transfection was carried out according to the instructions. After transfection, osteogenic induction medium was added to induce the induction of MSCs into osteoblasts. On the seventh day of osteoinduction, siRNA was transfected again for a second knockdown. The overexpression lentivirus was constructed by OBiO Technology (Shanghai, China). Mut-ALKBH5 contains a mutation of histidine to alanine at position 204. MSCs were incubated with the lentiviruses for 24 h at a multiplicity of infection of 30.

LY294002 and SC79 were purchased from Calbiochem and dissolved into dimethyl sulfoxide. MSCs were treated with 10 μM LY294002 and 4 µg/ml SC79 after siRNA or lentivirus transfection.

### m^6^A dot blot

Total RNA was extracted from the cells using TRIzol and the RNA concentration was determined by a NanoDrop 2000 (Thermo Fisher Scientific). The RNA concentration of different samples was adjusted to 250 and 500 ng/µl. Two microliters of RNA was spotted onto the nylon membrane (Sigma-Aldrich, GERPN1210B), followed by 1500 J ultraviolet crosslinking twice. One of the membranes was dyed in methylene blue solution for 30 min. After 30 min, the membrane was rinsed twice with ultrapure water and photographed. The other membrane was blocked with 5% nonfat dry milk dissolved in PBST (PBS with 0.1% Tween-20) for 1 h, followed by incubation with m^6^A antibody (1 : 1000, 202003, Sysy) for 14–16 h. Then, PBST was added to wash the membrane for 5 min, which was repeated three times. An horseradish peroxidase-conjugated AffiniPure goat anti-mouse IgG dilution (1 : 3000, BA1050, BOSTER) was added and the membrane was incubated at room temperature for 1 h. Then, PBST solution was added again to rinse the membrane three times. The protein signals were detected using chemiluminescent reagents (Millipore) according to the manufacturer’s instructions.

### m^6^A RNA methylation assay

This experiment was performed using the m^6^A RNA Methylation Assay Kit (ab185912, Abcam) according to the manual. In short, TRIzol was used to extract total RNA from the cells, the concentration was adjusted to 100 ng/μl after determining the RNA concentration, and 2 μl of RNA, 2 μl of negative control, and 2 μl of positive control were added to the well plate. Then, 100 μl of Developer Solution was added and the plate was incubated at room temperature for 6 min in the dark. Then, 100 μl of Stop Solution was added to stop the enzyme-linked reaction. The absorbance value was measured in a microplate reader (450 nm). The overall m^6^A methylation level was calculated according to the absorbance value and the formula used was as follows:

$${\mathrm{m}^6{\mathrm{A}}}\% = \frac{{\left( {\mathrm{Sample}}\;{\mathrm{OD}} - {\mathrm{NC}}\;{\mathrm{OD}} \right) \div {\mathrm{S}}}}{( {\mathrm{PC}}\;{\mathrm{OD}} - {\mathrm{NC}}\;{\mathrm{OD}}) \div {\mathrm{P}}} \times 100\%$$

where Sample OD is the OD value of sample well, NC OD is the OD value of negative control well, PC OD is the OD value of positive control well, S is total mass of RNA added to sample well, and P is the total mass of the positive control added to the positive control well.

### m^6^A-seq and RNA-seq

After using siRNA to interfere with the expression of ALKBH5 in MSCs, osteogenic differentiation was induced for 3 days. Total RNA was extracted using TRIzol reagent (Invitrogen). Approximately 50 µg of total RNA was subjected to isolation of poly(A) mRNA with poly-T oligo-attached magnetic beads (Invitrogen). After fragmentation, RNA was incubated with an m^6^A-specific antibody (No. 202003, Synaptic Systems, Germany) for immunoprecipitation. Then, eluted m^6^A-containing fragments and untreated input control fragments were converted to the final cDNA library in accordance with strand-specific library preparation by the dUTP method. Paired-end 2 × 150 bp sequencing was performed on an Illumina NovaSeq™ 6000 platform at LC-BIO Biotech, Ltd (Hangzhou, China).

### meRIP-qPCR and RIP-qPCR

The Magna RIP™ RNA-Binding Protein Immunoprecipitation Kit (Millipore) was used for the RIP assay. Briefly, 1 × 10^7^ cells were collected by RIP lysis buffer and then incubated with RIP buffer containing magnetic beads conjugated to anti-m^6^A (202003, sysy) and anti-ALKBH5 (ABE547, Merck Millipore) antibodies. Purified rabbit IgG was used as a negative control. Then, immunoprecipitated RNAs were isolated and purified for qPCR analysis to detect the presence of the target mRNA.

### Nuclear and cytoplasmic fractionation

This experiment was performed according to the instructions of the PARIS™ Kit (AM1921, Thermo Fisher). The cells were collected after digestion with trypsin. Cell fractionation buffer was added to the cells and the cells were then centrifuged. Then, Cell Disruption Buffer was added to lyse the nuclei. Then, the mixture was transferred to a filter column, preheated elution solution was added to dissolve the RNA after centrifugation, and the RNA concentration was measured. After reverse transcription to cDNA, qPCR was used to detect the expression of the target gene.

### RNA decay assay

After MSCs were transfected with siRNA, they were added to osteogenic induction medium and cultured for 3 days. Subsequently, 5 μg/ml actinomycin D was added. Total RNA at 0, 1, 2, and 4 h was extracted using TRIzol reagent (Invitrogen) and the relative expression was detected by qPCR according to a previously described method^39^.

### µCT and histomorphometric analyses

We selected 6-month-old male mice for bone mass analysis. µCT was used to analyze the bone structure of the femur. The collected bone tissues were fixed in 4% polyoxymethylene for 2 days and then stored in 70% ethanol at 4 °C before being processed. To analyze the trabecular bone, images were acquired at an effective pixel size of 9.56 μm, a voltage of 80 kV, a current of 500 μA, and an exposure time of 1500 ms in each of the 360 rotational steps. The BV/TV, bone surface area/BV, Tb. Th, Tb. N, and trabecular spacing were analyzed according to the guidelines^40^. Two-dimensional and three-dimensional bone structure image slices were reconstructed.

### H&E staining and immunohistochemical staining

The dissected femurs were fixed in 4% polyoxymethylene for 2 days and decalcified in 10% EDTA for 2 weeks before sectioning (5 μm). For H&E staining, the experiment was performed according to the manufacturer’s instructions (Beijing Solarbio Science & Technology Co, Ltd). For immunohistochemical staining, slides were treated with trypsin solution at 37 °C for 30 min for antigen retrieval and then incubated with rabbit anti-osteocalcin (1 : 500, Abcam, catalog number ab93876).

### Immunofluorescence staining

MSCs were seeded on sterile glass coverslips and osteogenic differentiation was induced for 10 days. Cells were fixed in 4% paraformaldehyde for 30 min and then 0.1% Triton X-100 was added for 15 min at room temperature. Normal goat serum (5%) was used to block cells for 30 min. A primary antibody against collagen I (1 : 500, Abcam, ab34710) was added and the cells were incubated overnight at 4 °C. Then, anti-rabbit IgG (1 :5 00, Cell Signaling Technology, 4413) was added and incubated for 1 h at room temperature. 4′,6-Diamidino-2-phenylindole (DAPI) was used to counterstain the nuclei. Thereafter, the samples were viewed under a laser scanning confocal microscope at wavelengths of 555 nm (red, collagen I) and 405 nm (blue, DAPI).

### Statistical analysis

The experiments in this study were independently repeated at least three times. SPSS 18.0 software (SPSS, Chicago, IL, USA) was used to analyze the experimental data and the results are expressed as the mean ± SD. Student’s *t*-test and one-way analysis of variance followed by the Bonferroni test was performed for statistical analyses. The bone mass of mice in the CKO and wild-type (WT) groups was compared using an unpaired two-tailed Student’s *t*-test. *P* < 0.05 was considered statistically significant; *P < 0.05, **P < 0.01, and ns represents no statistical significance.

## Supplementary information

Supplementary materials file

Supplementary figure 1

Supplementary figure 2

Supplementary figure 3

Supplementary figure 4

## Data Availability

All Seq data have been deposited into Sequence Read Archive database with the identifier PRJNA727434.
