# Supplementary material for: The N6-methyladenosine demethylase ALKBH5 negatively regulates the osteogenic differentiation of mesenchymal stem cells through PRMT6
Source: Cell Death Dis. 2021 Jun 4;12(6):578. doi: 10.1038/s41419-021-03869-4 (PMC8178363; doi:10.1038/s41419-021-03869-4)
Supplement: Supplementary file 1 — Supplementary materials file [file 41419_2021_3869_MOESM1_ESM.docx]

**Supplementary Table 1: Primers of the analyzed genes.**

| Species | Gene | | Forward primer (5’-3’) | Reverse primer (5’-3’) | |
| --- | --- | --- | --- | --- | --- |
| *Homo sapiens* | GAPDH  ALKBH5  ACTB  PRMT6  U6 | GGAGCGAGATCCCTCCAAAAT  CGGCGAAGGCTACACTTACG  CATGTACGTTGCTATCCAGGC  GGAGTCGGAGAAACCCCTG  CGCTTCGGCAGCACATATAC | | | GGCTGTTGTCATACTTCTCATGG  CCACCAGCTTTTGGATCACCA  CTCCTTAATGTCACGCACGAT  TGAAACGTCCGTGTCTTGCTC  TTCACGAATTTGCGTGTCAT |

**Supplementary Table 2: The siRNA sequences of the analyzed genes.**

| Gene Name | Sense (5’-3’) | Antisense (5’-3’) |
| --- | --- | --- |
| ALKBH5 siRNA-1  ALKBH5 siRNA-2  PRMT6 siRNA-1  PRMT6 siRNA-2  Negative control | GACUGUGCUCAGUGGAUAUTT  GCUUCAGCUCUGAGAACUATT  GCACCGGCAUUCUGAGCAUTT  CCAGGUGAAGCAGCACUAUTT  UUCUCCGAACGUGUCACGUTT | AUAUCCACUGAGCACAGUCTT  UAGUUCUCAGAGCUGAAGCTT  AUGCUCAGAAUGCCGGUGCTT  AUAGUGCUGCUUCACCUGGTT  ACGUGACACGUUCGGAGAATT |

**Supplementary Figure.**

Supplementary Figure 1. Generation of conditional Alkbh5 knockout mice. (A) Schematic representation of the conditional Alkbh5 knockout strategy. Exon 1 was deleted after cre-mediated recombination, resulting in translation termination. (B) Representative images of PCR genotyping.

Supplementary Figure 2. Whole-transcriptome m^6^A-seq and RNA-seq detection of ALKBH5 downstream regulatory genes. (A) Number of m^6^A peaks and genes. (B) Volcano map of differentially expressed genes. (C) KEGG pathway enrichment analyses of differentially expressed genes.

Supplementary Figure 3. PRMT6 is upregulated during osteogenic differentiation of MSCs. (A) The protein level of PRMT6 was determined by Western blotting (n=9). (B) Correlation of PRMT6 and Runx2 or SP7 during the osteogenic differentiation of MSCs (n=9). All data are presented as the means ± SDs. **p*< 0.05

Supplementary Figure 4. The ALKBH5-PRMT6 axis controls the activation of the AKT signaling pathway to modulate the osteogenesis of MSCs. (A) Protein levels of Runx2 and SP7 were determined by Western blot analysis after adding LY294002 while knocking down ALKBH5. (B) Western blot detection of Runx2 and SP7 after adding SC79 while overexpressing ALKBH5. (C) Western blot detection of Runx2 and SP7 after adding SC79 while knocking down PRMT6. All data are presented as the means ± SDs. **p*< 0.05, ***p*< 0.01. (n=3 independent experiments with three different MSC lines)
