# Supplementary figures and images for: The N6-methyladenosine demethylase ALKBH5 negatively regulates the osteogenic differentiation of mesenchymal stem cells through PRMT6
Source: Cell Death Dis. 2021 Jun 4;12(6):578. doi: 10.1038/s41419-021-03869-4 (PMC8178363; doi:10.1038/s41419-021-03869-4)

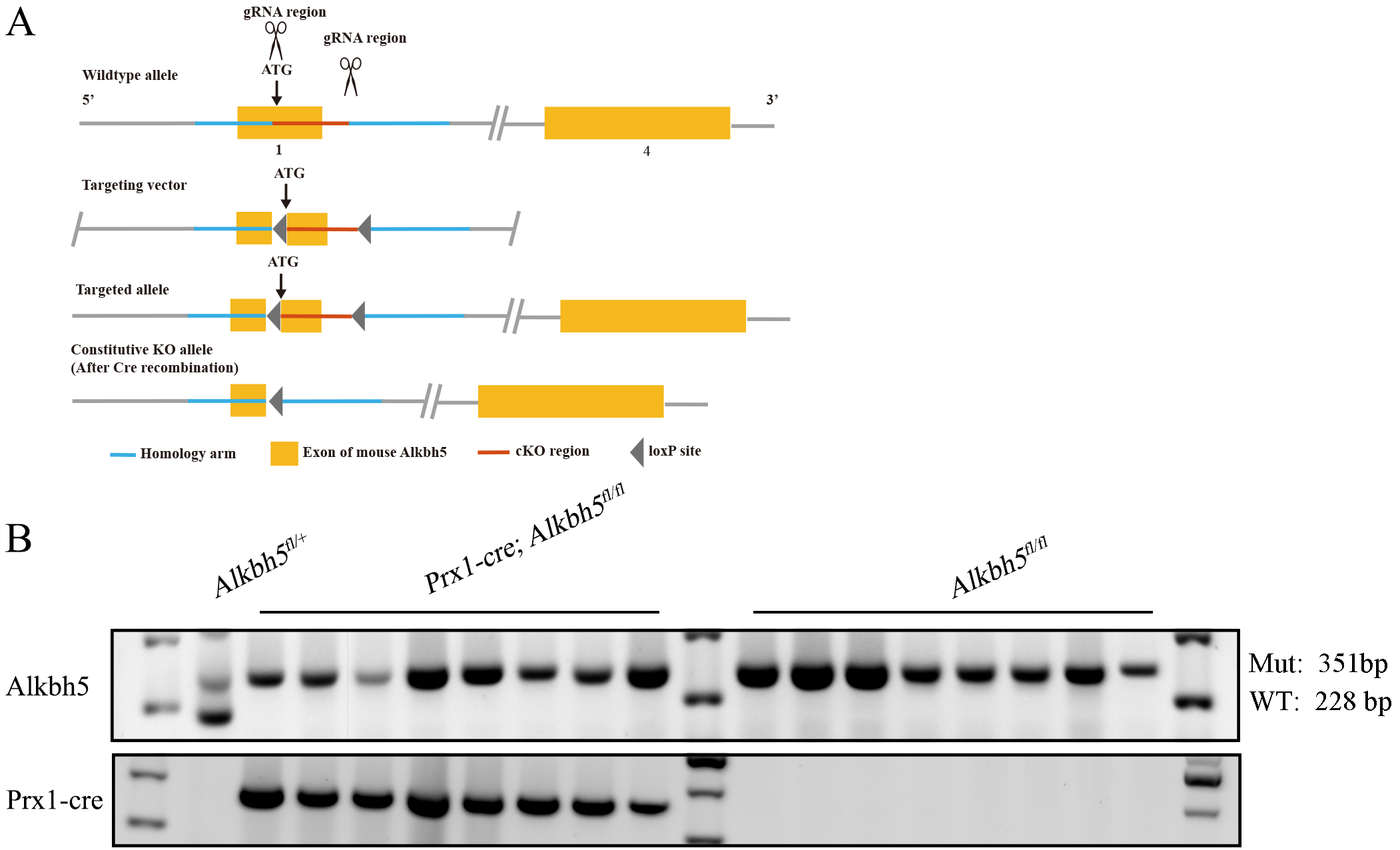

Supplement: Supplementary file 2 — Supplementary figure 1 [file 41419_2021_3869_MOESM2_ESM.tif]

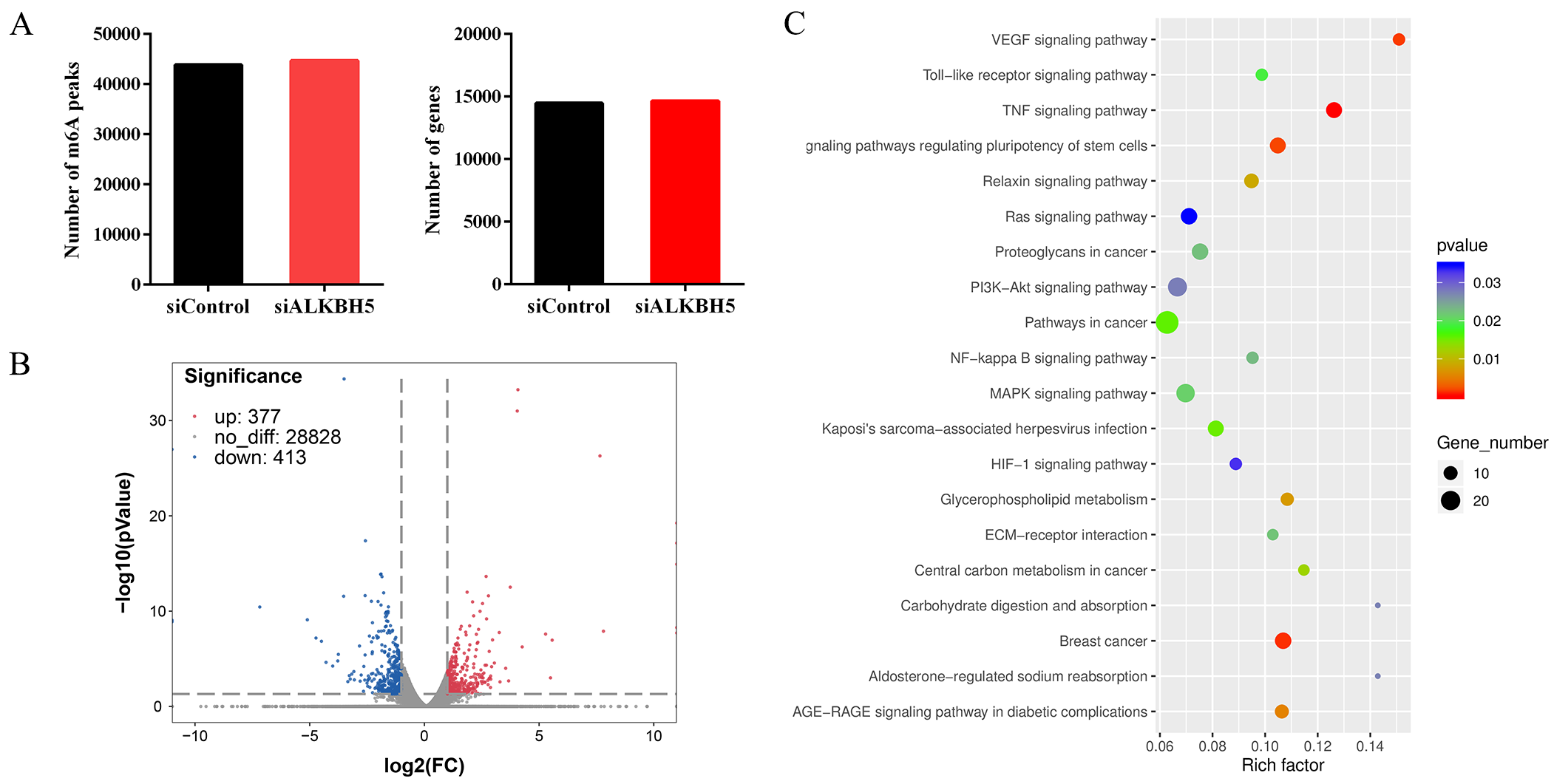

Supplement: Supplementary file 3 — Supplementary figure 2 [file 41419_2021_3869_MOESM3_ESM.tif]

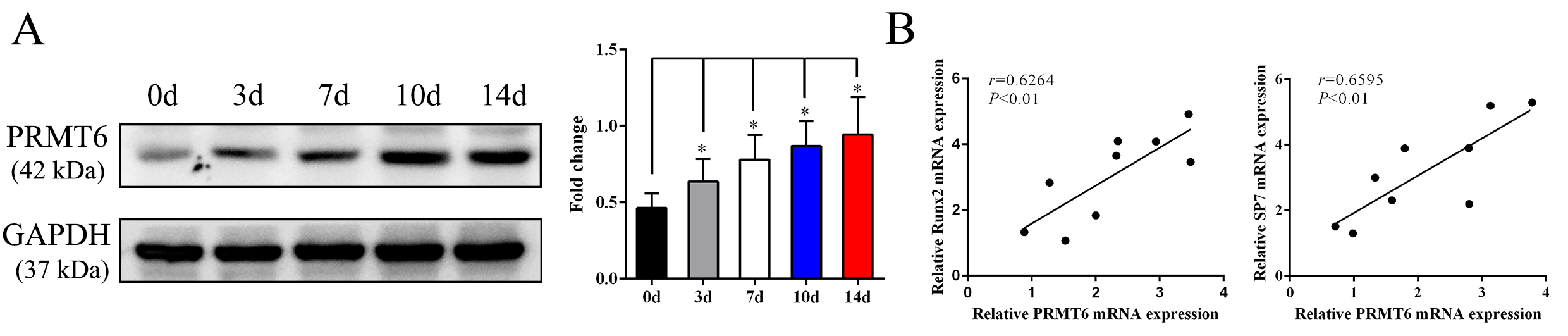

Supplement: Supplementary file 4 — Supplementary figure 3 [file 41419_2021_3869_MOESM4_ESM.tif]

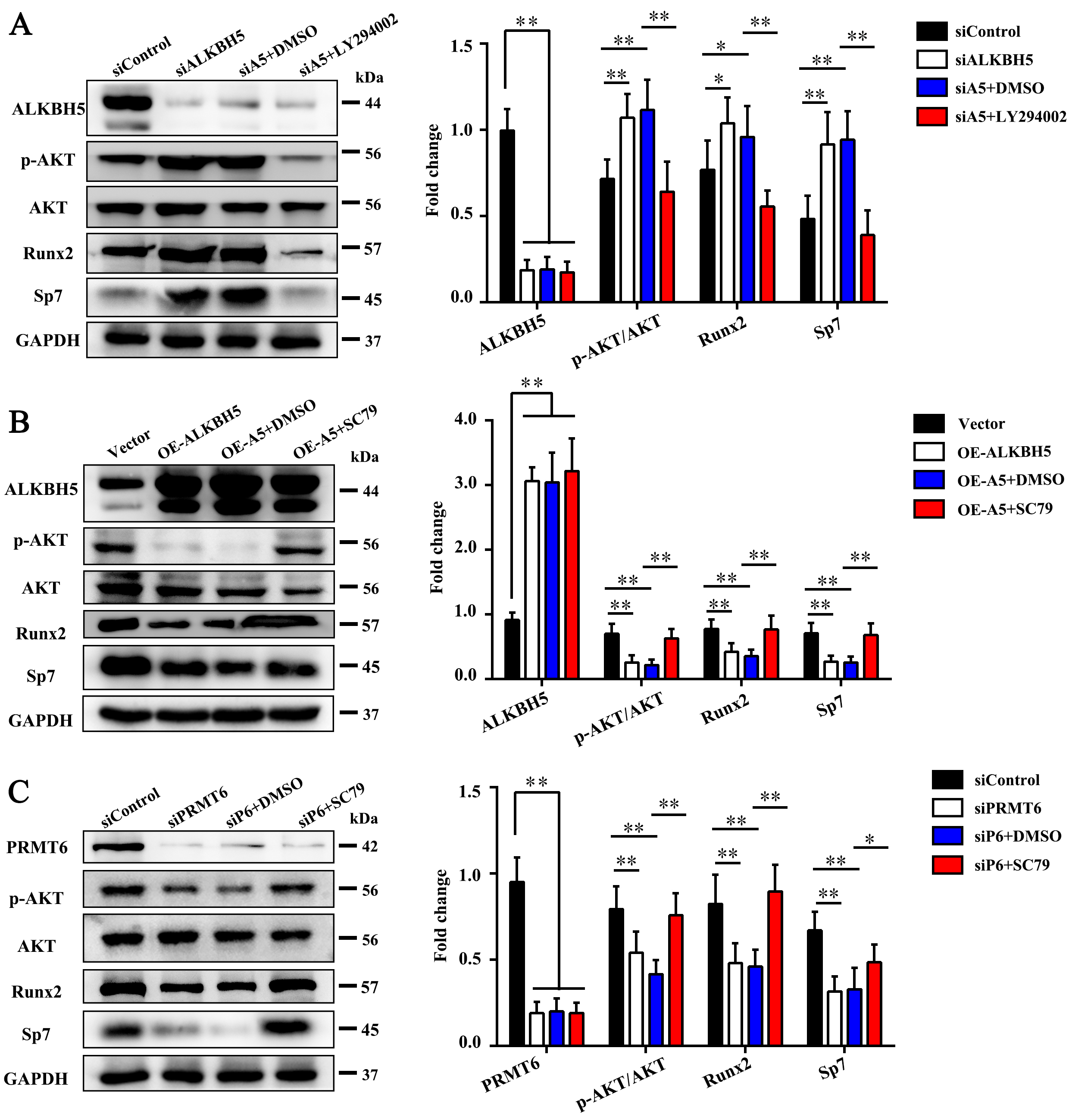

Supplement: Supplementary file 5 — Supplementary figure 4 [file 41419_2021_3869_MOESM5_ESM.tif]
